# Clinical efficacy and safety comparison of Watchman device versus ACP/Amulet device for percutaneous left atrial appendage closure in patients with nonvalvular atrial fibrillation: A study‐level meta‐analysis of clinical trials

**DOI:** 10.1002/clc.23956

**Published:** 2022-11-30

**Authors:** Sun Bing, Rui Rui Chen

**Affiliations:** ^1^ Department of Cardiology, Tang du Hospital Air Force Medical University Xi'an Shaanxi China

**Keywords:** Amulet, atrial fibrillation, left atrial appendage closure, nonvalvular atrial fibrillation, Watchman

## Abstract

Left atrial appendage occlusion is not inferior to oral anticoagulants in the prevention of stroke in several randomized controlled trials. However, the clinical efficacy and safety comparison of the Watchman and amplatzer cardiac plug (ACP)/Amulet devices for percutaneous left atrial appendage closure (LAAC) in patients with non‐valvular atrial fibrillation was controversial. A database search was conducted using PubMed, EMBASE, Cochrane Library, and Clinicaltrials.gov for trials that compared Watchman device vs ACP/Amulet device. The effective outcomes were stroke and systemic embolism. Safety outcomes were all‐cause death, cardiovascular death, and major bleeding. Device‐related complications included device‐related thrombus (DRT), peri‐device leaks (PDL > 5 mm). A total of 19 articles involving 6224 patients were included in the present study. The Watchman and ACP/Amulet groups comprised 3267 and 2957 patients, respectively. No statistically significant differences were detected in the stroke (odd ratio [OR]:1.24, 95% confidence interval [CI]: 0.92−1.67, *p* = .17, *I* ^2^ = 0), systemic embolism (OR:1.10, 95% CI: 0.51−2.35, *p* = .81, *I* ^2^ = 0%), all‐cause death (OR:0.97, 95% CI: 0.80−1.18, *p* = .77, *I* ^2^ = 1%), cardiogenic death (OR:0.99, 95% CI: 0.77−1.29, *p* = .96, *I* ^2^ = 0%), major bleeding (OR:1.18, 95% CI: 0.98−1.43, *p* = .08, *I* ^2^ = 25%). DRT (OR:1.48, 95% CI: 1.06−2.06, *p* = .02, *I* ^2^ = 0%) and PDL > 5 mm (OR:2.57, 95% CI: 1.63−4.04, *p* < .0001, *I* ^2^ = 0%) were significantly lower in ACP/Amulet group compared to Watchman group. The effective and safety outcomes were comparable between two groups. ACP/Amulet group had significantly lower rates of DRT and PDL > 5 mm than Watchman group.

## 1 INTRODUCTION

Atrial fibrillation (AF) is the most common among arrhythmia diseases. AF can lead to heart failure, stroke and other serious complications. Among these complications, stroke is the most serious condition that can lead to death and disability in AF patients. Oral anticoagulation (OAC) treatment is the most prevalent method of stroke prevention in AF patients. OAC can reduce the incidence of stroke by 64% and mortality by 26%.^1^, ^2^ However, long‐term anticoagulant therapy can increase the risk of bleeding in patients with high bleeding risk (HAS‐BLED ≥ 3). Therefore, an urgent need for an alternative treatment to reduce the risk of bleeding is required, and left atrial appendage closure (LAAC) becomes the best nonpharmacological treatment option.

The Watchman device (Boston Scientific) is a left atrial appendage device approved by the Food and Drug Administration (FDA) in the United States and is highly used. Based on the PROTECT AF and PREVAIL trials, the Watchman device was found to be noninferior in reducing stroke and systemic embolism events in patients with nonvalvular AF (NVAF) compared to OAC.^3^, ^4^ The 5‐year follow‐up showed that LAAC reduced the incidence of cardiac death and hemorrhagic stroke in NVAF patients but without statistically significantly increasing the risk of ischemic stroke.^5^ Several observational trials in nations other than the USA have demonstrated the effectiveness and safety of the amplatzer cardiac plug (ACP)/Amulet device and the Amulet is now an FDA approved LAAC device.^6^

However, the results from studies which directly compared the efficacy and safety of the Watchman and ACP/Amulet devices for NVAF patients were contradictory.^7^, ^8^, ^9^, ^10^, ^11^, ^12^, ^13^, ^14^, ^15^, ^16^, ^17^, ^18^, ^19^, ^20^, ^21^, ^22^, ^23^, ^24^, ^25^ Therefore, a meta‐analysis was performed in this study to compare the safety and efficacy of the two devices.

## 2 METHODS

### 2.1 Inclusion and exclusion criteria

The inclusion criteria: (1) Any clinical trials that included patients who suffered from NVAF with high stroke or bleeding risk and had undergone LAAC with Watchman device or ACP/Amulet device; (2) Studies reported any of effective or safety outcomes after LAAC. The exclusion criteria: Animal experiments, case reports, reviews, meta‐analyses, conference proceedings without a full manuscript, and trials that did not directly compare Watchman device versus ACP/Amulet device were excluded.

### 2.2 Intervention measures and outcomes

The Watchman or ACP/Amulet devices were used for left atrial appendage occlusion in patients. The effective outcomes were stroke and systemic embolism. Safety outcomes were all‐cause death, cardiovascular death, and major bleeding. Device‐related complications included device‐related thrombus (DRT), peri‐device leaks (PDL > 5 mm).

### 2.3 Search strategy

PubMed, EMBASE and Cochrane Library databases were searched automatically using the keywords “Atrial Fibrillation; Left atrial appendage closure; Left atrial appendage occlusion; Amplatzer Cardiac plug; Amplatzer; Watchman device; Watchman FLX.” Finally, the newly published and unincluded literature was searched manually. Two researchers independently screened the literature, submitted the data and cross‐checked it. In case of any disagreement, a third researcher was assigned to assist in the final decision. The extracted data included (1) author names and publication year; (2) basic characteristics of the research object; (3) follow‐up process; (4) all outcome index data. The Cochrane evaluation tool was used in RCT trials, and Newcastle‐Ottawa scale (NOS) scale was used in non‐RCT trials to evaluate the quality of the included literature.^24^, ^25^ The retrieval duration was from inception to July 1, 2022.

### 2.4 Statistical methods

RevMan 5.4 and Stata 17.0 was applied in statistical meta‐analysis. The odds ratio (OR) represented the effect index because the outcome index was a dichotomous variable, and a point estimation of 95% confidnce interval [CI] was given for each index. For every indicator, point valuation or 95% CI was prescribed. *p* < 0.05 were considered statistically significant. We evaluated statistical heterogeneity using Cochran's Q test (*p* < .05) and Higgins *I* ^2^ statistics. The fixed effect model was used in the meta‐analysis when *p* > .05 and *I* ^2^ < 0.5. Potential risk factors on outcomes were assessed by meta‐regression. Leave‐one‐out sensitivity analysis was performed to assess the contribution of each study to the pooled OR of the outcomes. Publication bias was analyzed by contour enhancement funnel plot and Egger's regression.

## 3 RESULTS

### 3.1 Literature screening

**Figure 1:**
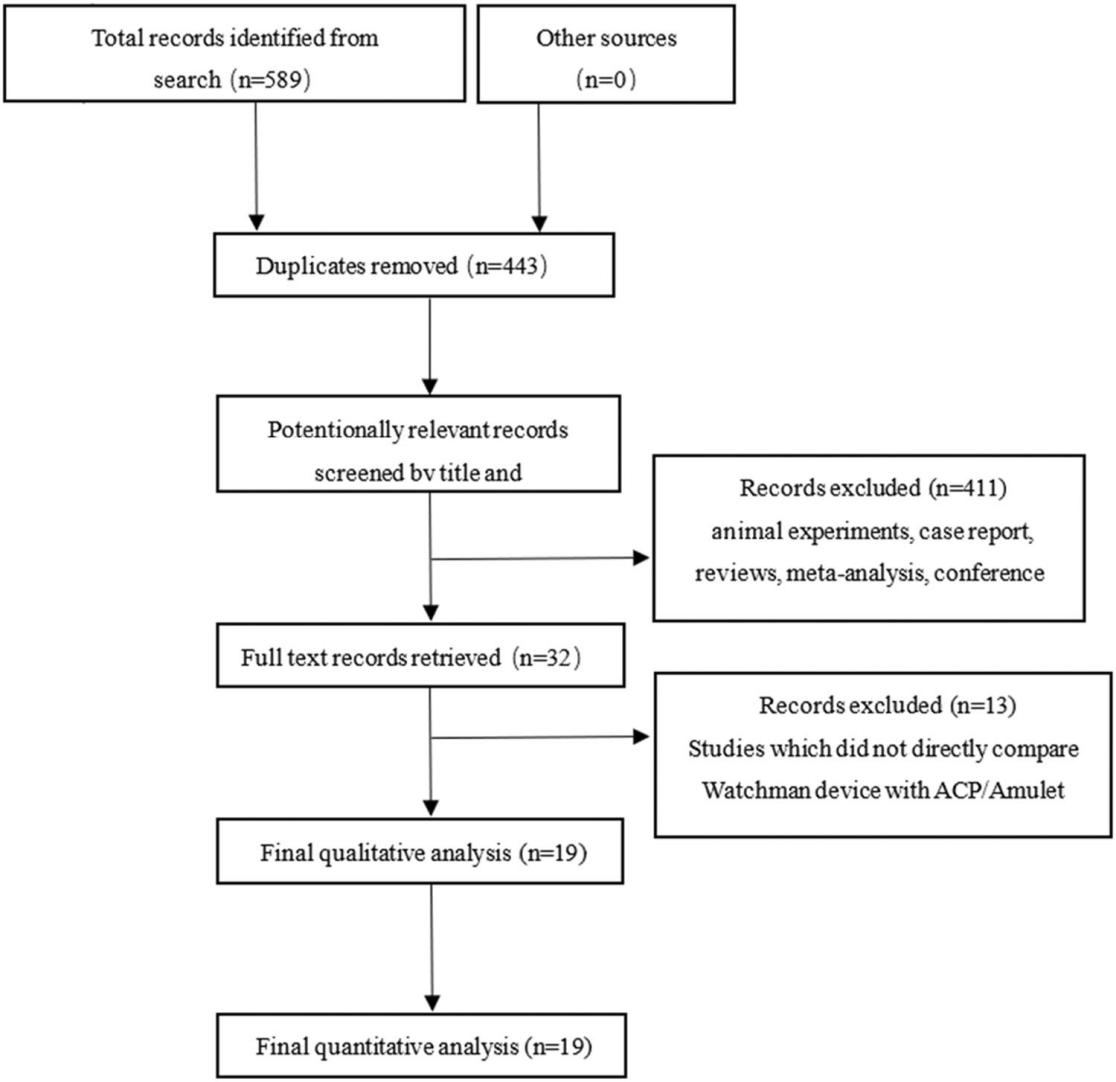
PRISMA flow diagram

A total of 589 studies were retrieved from PubMed, EMBASE, and Cochrane Library databases, and 443 articles remained after removing duplicates. In total, 411 articles were excluded by reading titles and abstracts (including reviews, animal experiments, meta‐analyses, case reports, and meeting minutes), 13 articles were further removed by reading the full text (13 articles did not directly compare the efficacy and safety of Watchman vs. ACP/Amulet), and finally 19 articles were included in qualitative and quantitative studies (Figure 1).

### 3.2 Base data and procedural characteristics

Authors, publication years, sample size, CHA2DS2‐VASC Score, HAS‐BLED Score, antithrombotic strategy and follow‐up time are illustrated in Table 1.

**Table 1:** Baseline data and procedural characteristics

Author	Year	Design	Sample size		CHA2DS2‐VASC Score(Mean ± SD)		HAS‐BLED Score		Anti‐thrombotic therapy	Follow‐up time
			Watchman	ACP/Amulet	Watchman	ACP/Amulet	Watchman	ACP/Amulet		
Chun	2013	Non‐RCT	40	40	4.1 ± 1.5	4.5 ± 1.8	3.1 ± 1.1	3.1 ± 1.2	OAC or DAPT 6 weeks, switch to SAPT	12 months
Cruz	2013	Non‐RCT	10	21	3.6 ± 0.8	3.7 ± 0.8	4.7 ± 1.3	4.6 ± 1.3	DAPT 3 months switch to SAPT	3 months
Gafoor	2013	Non‐RCT	26	27	5.3 ± 1.4	5.0 ± 1.5	NR	DAPT 3−6 months	12 months	
Kim	2016	Non‐RCT	46	50	4.1 ± 1.7	3.6 ± 1.6	2.8 ± 1.2	2.7 ± 1.3	OAC or DAPT 6 weeks, switch to DAPT	22 months
Figini	2016	Non‐RCT	66	99	3.8 ± 1.6	4.0 ± 1.7	3.4 ± 1.3	3.7 ± 1.5	DAPT 1−3 months switch to SAPT	15 months
Fastner	2018	Non‐RCT	154	35	4.5 ± 0.1	4.0 ± 1.4	3.6 ± 0.2	3.7 ± 1.0	OAC + SAPT or DAPT 6 months, switch to DAPT	6 months
Chen	2019	Non‐RCT	36	74	3.9 ± 1.5	3.6 ± 1.5	3.8 ± 1.0	3.9 ± 1.1	OAC or DAPT 6 months	6 months
Cheung	2019	Non‐RCT	67	77	3.9 ± 1.7	3.8 ± 1.4	2.7 ± 1.1	2.8 ± 0.9	OAC or DAPT 6 weeks, switch to SAPT	28 months
Jakob	2020	Non‐RCT	278	340	4.4 ± 1.5	4.6 ± 1.6	3.9 ± 1.0	3.9 ± 1.2	5.2% OAC, 5.5% DAPT, 89.3% SAPT	12 months
Caroling	2020	Non‐RCT	266	266	4.5 ± 1.7	4.5 ± 1.5	3.2 ± 1.0	3.2 ± 1.0	OAC or DAPT 6 months switch to SAPT clopidpgrel	30 months
Davtyan	2020	Non‐RCT	108	92	3.5 (3−5)	4 (3–5)	3 (2−3)	3 (3−3)	NOAC or NOAC + ASA or DAPT + Warfarin	12 months
Chiu	2021	Non‐RCT	56	56	4.2 ± 1.4	3.9 ± 1.8	3.5 ± 1.7	3.3 ± 1.9	OAC + SAPT or DAPT	28 months
Lakkireddy	2021	RCT	944	934	4.7 ± 1.4	4.5 ± 1.3	3.3 ± 1.0	3.2 ± 1.0	DAPT or OAC + SAPT 9 months switch to SAPT	18 months
Mansour	2021	RCT	25	26	3.9 ± 1.27	3.9 ± 1.16	4.2 ± 0.9	4.1 ± 1.2	NR	12 months
Mohammed	2021	Non‐RCT	113	113	4 (3−5)	4 (3–5)	3 (2−3)	3 (2−3)	OAC or DAPT 6‐12weeks, switch to SAPT	6‐8months
Rinodivic	2021	Non‐RCT	99	125	3.5	3.3	4.1	3.9	OAC or DAPT 3 months switch to SAPT	4 years
Teiger	2021	Non‐RCT	378	434	4.6 ± 0.1		3.2 ± 0.05		22.1% OAC, SAPT 33.2%, DAPT 40.4%	16 months
Galea	2022	RCT	110	111	4.4 ± 1.4	4.2 ± 1.4	3.2 ± 1.0	3.1 ± 0.8	OAC + SAPT or DAPT 3 months switch to SAPT	45 days
Kretzler	2022	Non‐RCT	389	93	4.1 ± 1.5	4.2 ± 1.5	3.6 ± 1.1	3.3 ± 1.2	OAC or DAPT 6 weeks, switch to SAPT	6 weeks

Abbreviation: NOAC: novel oral anticoagulants.

### 3.3 Quality assessment

**Figure 2:**
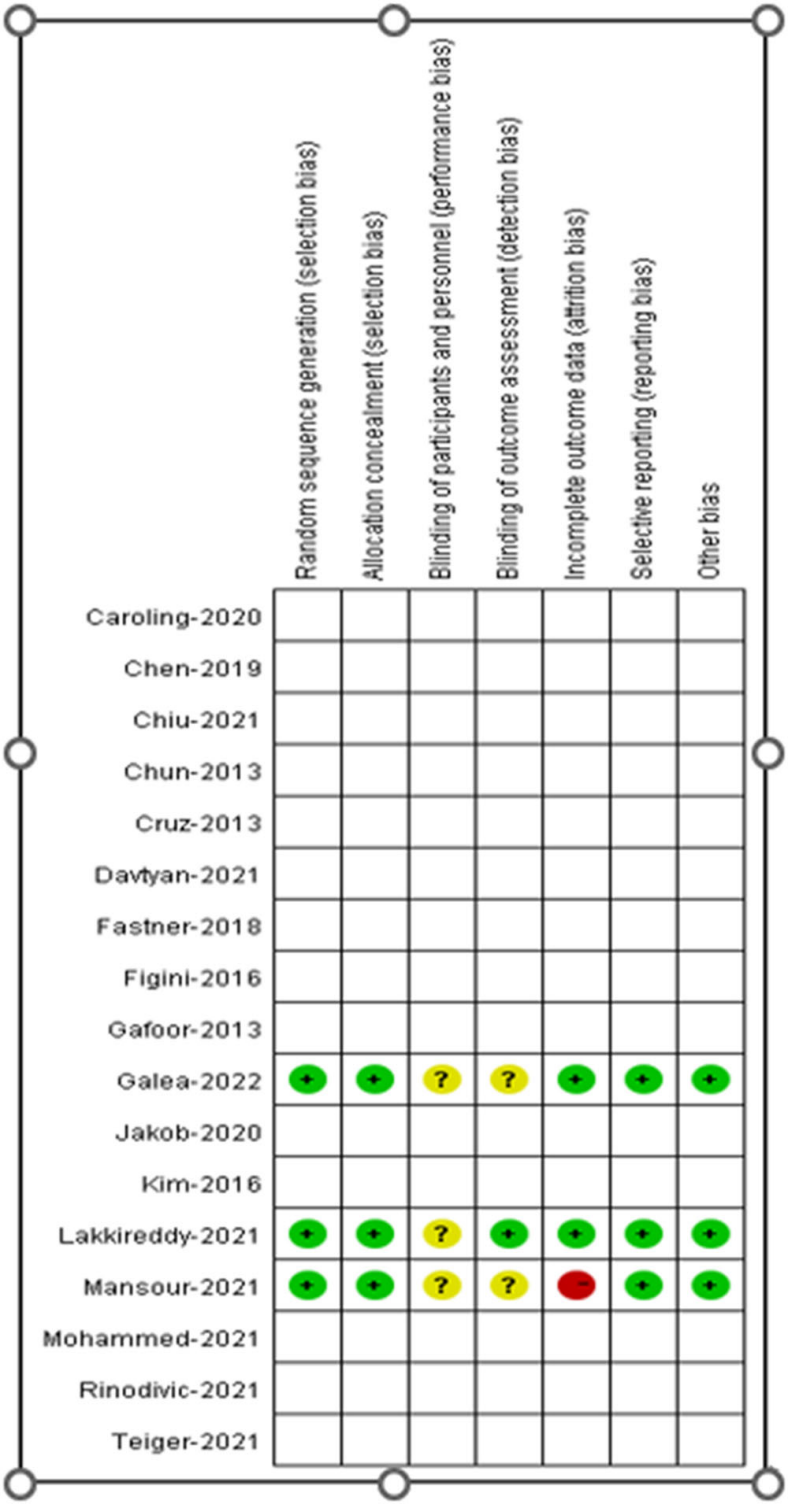
Quality assessment

A total of 19 articles were investigated in the present study, including three RCTs and sixteen non‐RCTs. The Cochrane evaluation tool was employed for the RCT subgroup.^26^ NOS scales were applied for the non‐RCTs to evaluate the quality of the covered literature.^27^ The quality evaluation of the RCT literature reflected high quality (Figure 2). The NOS scale evaluation results of the non‐RCTs are illustrated in Table 2.

**Table 2:** Quality assessment

Author and year	Selection of case (4 scores)				Comparison between Definition and diagnosis of cases	methods of exposure assessment (4 scores)		
	Definition and diagnosis of cases	Representativeness of cases	Control selection	Definition of control		Representativeness of cases	Same method of ascertainment for cases and controls	Investigation No response rates
Chun‐2013	√			√	√	√	√	√
Cruz‐2013	√		√	√	√	√	√	√
Gafoor‐2013	√	√	√	√	√	√	√	√
Kim‐2016	√		√	√	√√	√	√	√
Figini‐2016	√	√	√	√	√	√	√	√
Fastner‐2018	√	√	√	√	√√	√	√	√
Chen‐2019	√	√	√	√	√	√	√	√
Cheung‐2019	√	√	√	√	√	√	√	√
Jakob‐2020	√	√	√	√	√√	√	√	√
Caroling‐2020	√			√	√	√	√	√
Davtyan‐2020	√	√	√	√	√√	√	√	√
Chiu‐2021	√	√	√	√	√√	√	√	√
Mohammed‐2021	√	√	√	√	√√	√	√	√
Rinodivic‐2021	√	√	√	√	√	√	√	√
Teiger‐2021	√	√					√	√
Kretzler‐2022	√	√	√	√	√	√	√	

### 3.4 Outcomes

#### 3.4.1 Effective endpoints

No significant differences were observed in stroke and systematic embolism. Stroke: Watchman 3.0% versus ACP/Amulet 2.7% (OR: 1.24, 95% CI: 0.92−1.67, *p* = .17, *I* ^2^ = 0%) (Supporting Information: Figure 1). Systematic embolism: Watchman 0.5% versus ACP/Amulet 0.5% (OR: 1.10, 95% CI: 0.51−2.35, *p* = .81, *I* ^2^ = 0%) (Supporting Information: Figure 2).

#### 3.4.2 Safety endpoints

No significant differences were observed in all‐cause death, cardiogenic death and major bleeding. All cause death: Watchman 7.5% versus ACP/Amulet 8.5% (OR: 0.97, 95% CI: 0.80−1.18, *p* = .77, *I* ^2^ = 1%) (Supporting Information: Figure 3). Cardiogenic death: Watchman 3.6% vs ACP/Amulet 4.2% (OR: 0.99, 95% CI: 0.77−1.29, *p* = .96, *I* ^2^ = 0%) (Supporting Information: Figure 4). Major bleeding: Watchman 9.6% versus ACP/Amulet 9.0% (OR: 1.18, 95% CI: 0.98−1.43, *p* = .08, *I* ^2^ = 25%) (Supporting Information: Figure 5).

#### 3.4.3 Device‐related complications

There were statistically significant differences detected in DRT Watchman 3.3% versus ACP/Amulet 2.2% (OR: 1.48, 95% CI: 1.06–2.06, *p* = .02, *I* ^2^ = 0%) and PDL > 5 mm Watchman 2.4% versus ACP/Amulet 1.0% (OR: 2.57, 95% CI: 1.63–4.04, *p* < .0001, *I* ^2^ = 0%) (Supporting Information: Figure 6−7).

#### 3.4.4 Sensitivity analysis

Leave‐one‐out sensitivity analyses was used by Stata 17.0. All results were consistent with main analysis.

#### 3.4.5 Publication bias

Publication bias was analyzed for any major adverse events by using the contour enhancement funnel plot and shear complement method. Imputed studies obtained = 3 (Supporting Information: Figure 8). Our study had minor publication bias.

## 4 DISCUSSION

The purpose of this study was to compare the efficacy and safety of the Watchman device vs the ACP/Amulet device for NVAF patients with high risk of stroke or bleeding. There were three main findings in our study:

(1) No statistical differences were detected between the Watchman and the ACP/Amulet group in terms of stroke, systematic embolism.
(2) The all‐cause death and cardiogenic death were similar between two groups. and major bleeding. Watchman group had a potential trend of higher occurrences of major bleeding than ACP/Amulet group, though it did not have statistically significant difference.
(3) The Watchman group had a significantly higher incidence of DRT (3.3% vs. 2.2%) and PDL > 5 mm (2.4% vs. 1.0%) than ACP/Amulet group.

Effective and safety outcomes were comparable between two groups, while major bleeding was slightly lower in ACP/Amulet group (9.0% vs. 9.6%). Patients who were discharged on OACs were lower in ACP/Amulet group, which may result in lower major bleeding.^21^

Although DRT is uncommon, it was related to a threefold higher stroke and risk of having systemic embolism.^28^ The incidence of DRT in this study was 3.3% in the Watchman group and 2.2% in the ACP/Amulet group, which were consistent with existing studies.^6^, ^29^ The risk of DRT tended to be higher in Watchman group than ACP/Amulet group (3.3% vs. 2.2%). History of TIA or stroke, permanent AF, vascular disease, female sex, older age, smoking, greater LAA diameter or orifice width, reduced left ventricular ejection fraction, and presence of spontaneous echocardiography contrast were risk factors of DRT.^28^, ^29^ Unfortunately, due to the unavailability of individual data, we cannot make a multivariable analysis of the above‐mentioned risk factors. In the future, a predictive DRT model is needed guide us in selecting anticoagulant strategies. PDL is another potential factor that affects the effective endpoints. The incidence of PDL is between 5% and 32%, and PDL may be associated with the incidence of major adverse cardiac events. Currently, the generally accepted treatment plan is cases with PDL < 3 mm are not associated with embolism events and do not require special treatment, while PDL > 5 mm requires OAC treatment. However, recent studies revealed that small leaks (0–5 mm) after the LAAC procedure were related to a modestly higher occurrence of thromboembolic and bleeding events, and on the opposite side, larger leaks (>5 mm) were not related to adverse events.^30^, ^31^ In this study, the incidence of PDL > 5 mm was significantly higher in Watchman group than in ACP/Amulet group (2.7% vs. 1%). The Amulet occlude has a dual‐seal mechanism and consists of a lobe and a disc connected by a central waist with polyester patches sewn into both the lobe and disc to facilitate effective occlusion. This design may help to overcome the limitations of a single‐seal mechanism, including but not limited to short LAA length, proximal lobes near the ostium, and very large ostia.^32^

Although LAAC has been widely used in many centers, there are still some problems that need to be solved. First, more specific antithrombotic strategies are required. Warfarin had been widely used in the first 45 days for the postimplant anticoagulation after LAAC for preventing device‐related complications in previous pivotal trials,^3^, ^4^ while novel oral anticoagulants (NOAC) was used more frequently in resent studies.^33^ Nowadays, neither the American College of Cardiology/American Heart Association (ACC/AHA) nor the European Society of Cardiology (ESC) guidelines had specific recommendations on NOAC or warfarin at discharge after LAAC and the choice is left at the clinician's discretion.^1^, ^2^ The Chinese Society of Cardiology (CSC) of the Chinese Medical Association recommends using OAC monotherapy for patients with high bleeding risk (30 days after LAAC) (HAS‐BLED ≥ 3 points), while OAC + aspirin was used for those with low bleeding risk (HAS‐BLED < 3 points).^34^ However, these recommendations were based on small clinical trials. Second, the efficacy and safety of the new generation device need to be verified. The Watchman FLX is a new generation product. The PINNACLE FLX study included 400 patients who underwent LAAC with Watchman FLX. The incidence of DRT was 1.75% at 12 months follow‐up and the incidence of PDL > 5 mm was 0%.^35^ The new generation device Watchman FLX is a new option to look forward for further applications. Watchman FLX achieved a near 100% implantation success and a substantial decrease in the occurrence of periprocedural complications as compared to Amplatzer Amulet occlusion.^36^ Watchman FLX also had a lower DRT at 45 days^37^ and a higher sealing rate at 3 months as compared to the Watchman device in small sample size of clinical trials.^38^ Watchman FLX is the commonly implanted device, but it has not been widely used in most developing countries. Therefore, this study does not include the Watchman FLX. A comparison of the Amulet to the FLX device would be of significantly more interest in the future.

This study also had the following limitations. First, this study was a study‐level meta‐analysis, and the original data of all included studies was not obtained. The influence of the baseline data on the results of this study is unknown. Second, the duration of the included studies was long and the experience of the operators may influence the results. Additionally, the antithrombotic strategies of the included studies were different, and the influence of antithrombotic strategies on the results could not be analyzed. Finally, the follow‐up time of the included studies ranged from 3−48 months, and different follow‐up times can affect the effective and safety endpoints.

## 5 CONCLUSION

Similar incidences of effective and safety outcomes were observed between the Watchman and ACP/Amulet groups. The ACP/Amulet have lower DRT and peridevice leaks (PDL > 5 mm). The absolute risk reduction was 1.1% and 1.4% with the Amulet device about DRT and PDL > 5 mm. Although the differences of DRT and PDL > 5 mm between the devices were statistically significant, in large sample sizes of our real world, every day clinical practice, this is insignificant. However, this conclusion is based on data from present studies, which needs to be further verified.

## Supporting information

Supporting information.

## Data Availability

The original contributions presented in the study are included in the article/Supporting Information Material, further inquiries can be directed to the corresponding authors.
