# Supplementary material for: Clinical efficacy and safety comparison of Watchman device versus ACP/Amulet device for percutaneous left atrial appendage closure in patients with nonvalvular atrial fibrillation: A study‐level meta‐analysis of clinical trials
Source: Clin Cardiol. 2022 Nov 30;46(2):117–25. doi: 10.1002/clc.23956 (PMC9933112; doi:10.1002/clc.23956)

**Supplementary file**

Supplementary Figure 1. Stroke

Supplementary Figure 2. Systematic embolism

Supplementary Figure 3. All-cause death

Supplementary Figure 4. Cardiogenic death

Supplementary Figure 5. Major bleeding

Supplementary Figure 6. Device-related thrombus (DRT)

Supplementary Figure 7. Peri-device leaks (PDL<5mm)

Supplementary Figure 8. Publication bias

Supplementary Figure 1. Stroke


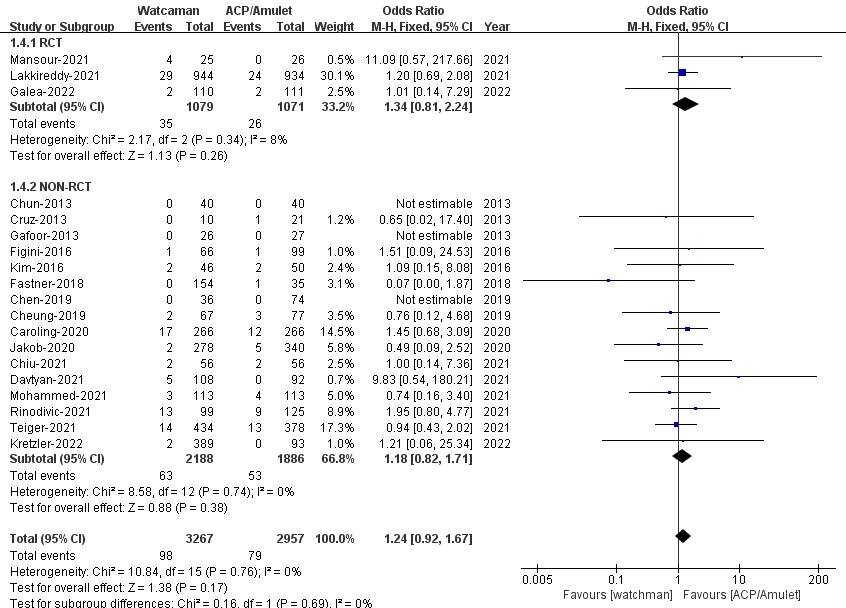


Supplementary Figure 2. Systematic embolism


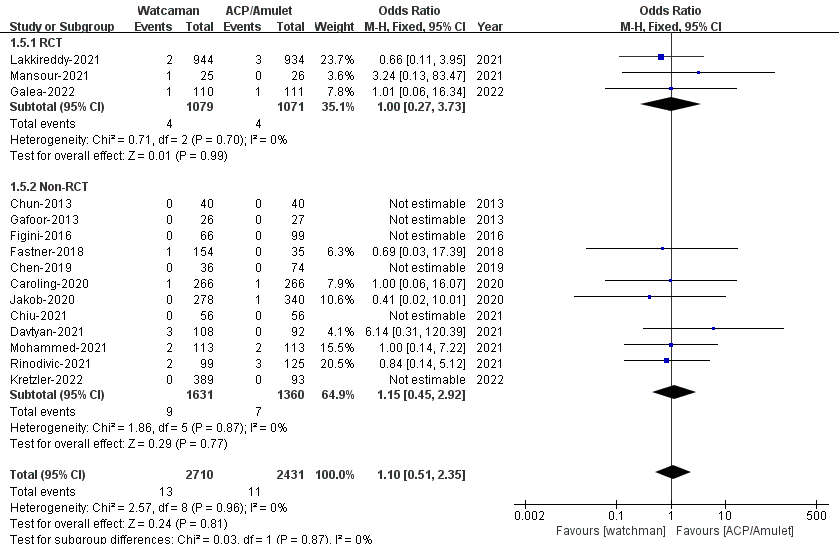


Supplementary Figure 3. All-cause death


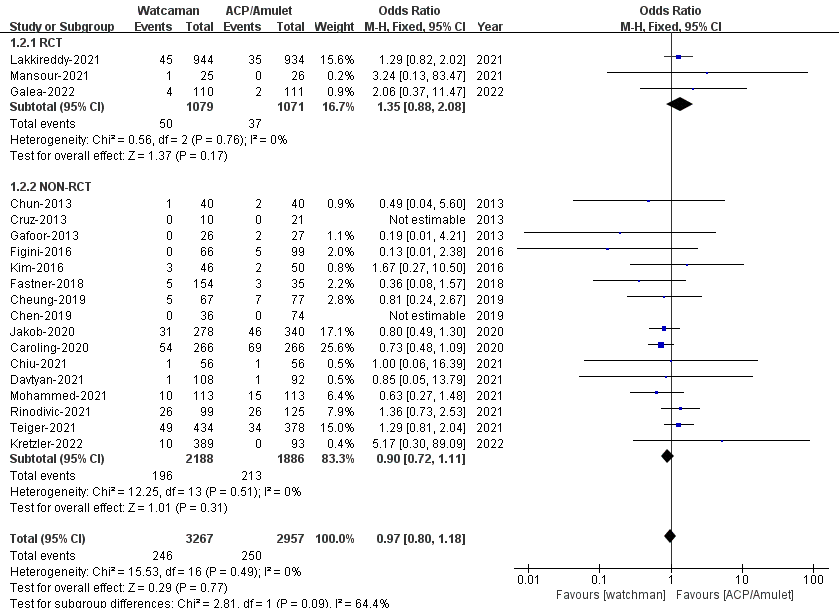


Supplementary Figure 4. Cardiogenic death


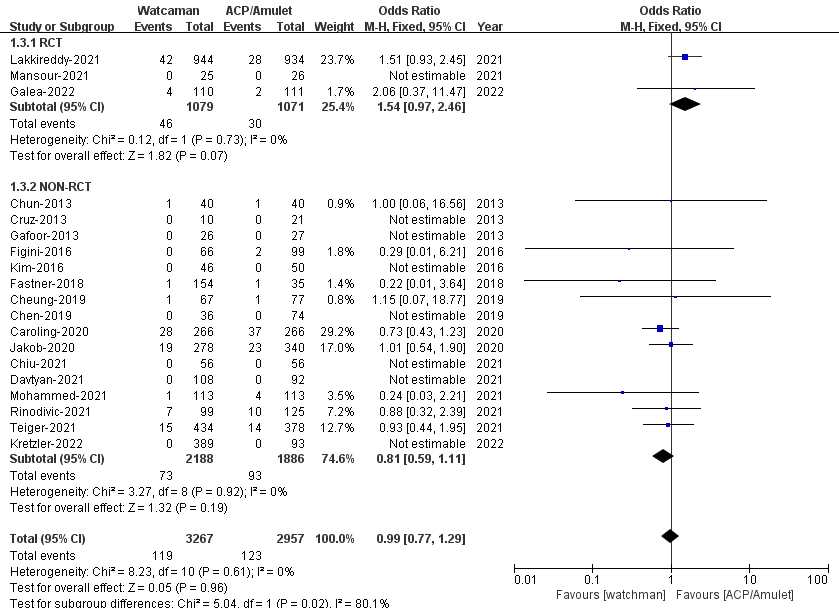


Supplementary Figure 5. Major bleeding


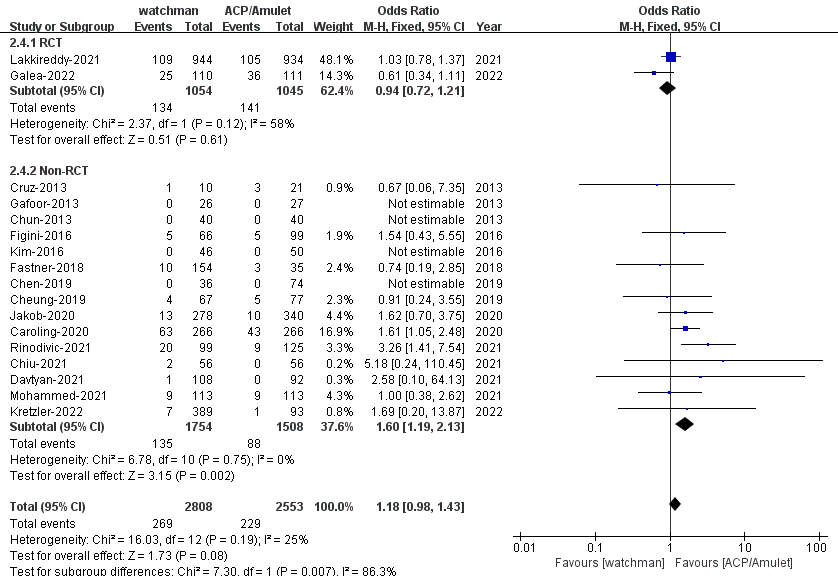


Supplementary Figure 6. Device-related thrombus (DRT)


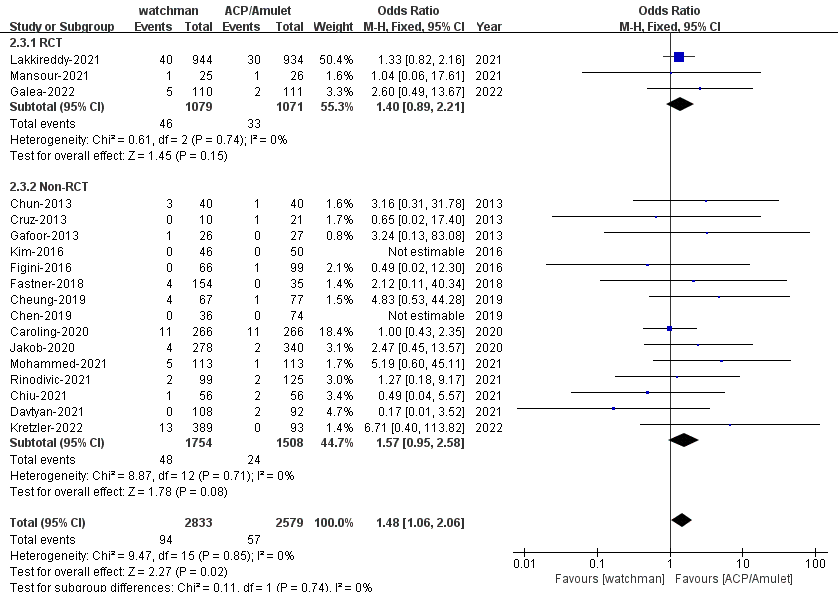


Supplementary Figure 7. Peri-device leaks (PDL>5mm)


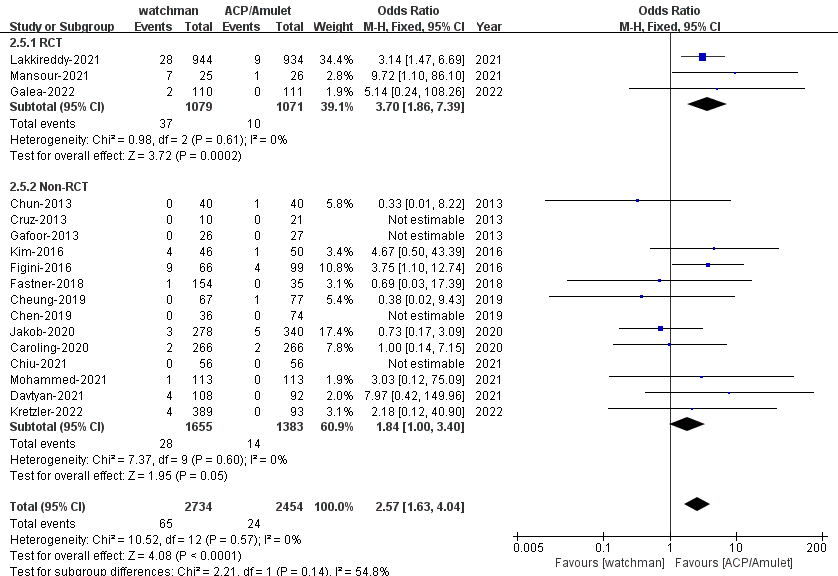


Supplementary Figure 8. Publication bias


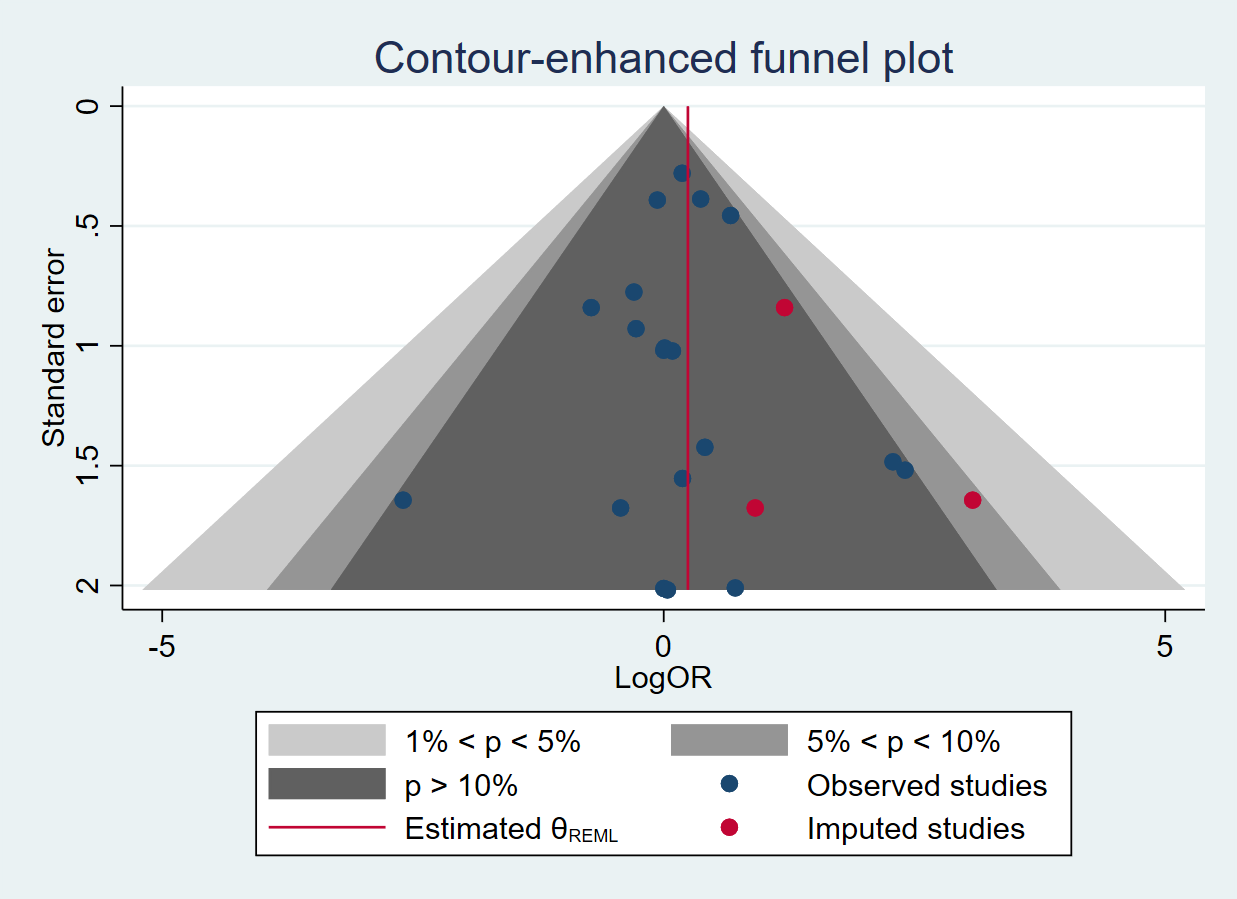

Supplement: Supplementary file 1 — Supporting information. [file CLC-46-117-s001.docx]
